# Influence of Barley Grain Architecture on Colonic Fermentation and Metabolism: A Narrative Review

**DOI:** 10.3390/foods15213799

**Published:** 2026-10-23

**Authors:** Sukanya Chakraborty, John Monro, Michael W. Taylor, Nicole C. Roy, Anne-Louise M. Heath, Pramod K. Gopal

**Affiliations:** 1 Department of Human Nutrition, University of Otago, Dunedin 9054, New Zealand; sukanya.chakraborty@plantandfood.co.nz (S.C.); anne-louise.heath@otago.ac.nz (A.-L.M.H.); 2 New Zealand Institute for Bioeconomy Science Limited, Palmerston North 4442, New Zealand; john.monro@plantandfood.co.nz; 3 Riddet Institute, University Avenue, Fitzherbert, Palmerston North 4474, New Zealand; 4 School of Biological Sciences, University of Auckland, Auckland 1010, New Zealand; mw.taylor@auckland.ac.nz

**Keywords:** barley, β-glucan, resistant starch, particle size, gut microbiota, microbial fermentation, short-chain fatty acids, food structure, enteroendocrine signalling

## Abstract

The human health effects of barley are considered to arise mainly from its undigested carbohydrates, yet how its grain composition translates to physiological effects is not fully understood. Most studies focus on total dietary fibre content rather than on how carbohydrates are arranged within the grain, leaving open questions about how barley grain structure may influence fermentation within the colon. The organisation of cell walls, starch granules, and the surrounding non-starch polysaccharides decides what escapes digestion in the gastrointestinal tract and is therefore accessible to gut microbes during colonic fermentation. This narrative review explores how barley grain morphology may influence the onset, duration, and location of gut fermentation, and how this may affect metabolic pathways. Therefore, we propose hypotheses linking processing formats to fermentation patterns and host signalling, aiming to address clear gaps in our understanding of this area. Closing these gaps could enable processing strategies that modulate colonic fermentation dynamics and target specific colonic responses.

## 1. Introduction

Consumption of barley (*Hordeum vulgare*) is often associated with health benefits in humans, such as improved cardiovascular health [1], but the mechanisms underlying these effects are not entirely clear. Most studies have examined barley’s non-starch polysaccharides (NSPs), especially β-glucans, and their roles in glucose homeostasis, gastrointestinal signalling of satiety hormones, and changes in the gut microbiota [2,3]. Studies show that the viscosity and amount of β-glucan are key factors in postprandial glucose and fermentation outcomes [4]. However, most work has focused on isolated or dissolved fibre, and not on how the grain’s morphology might affect whether β-glucan is trapped inside cell walls or easily available. Barley’s structure can be described at three levels: the grain’s layers, the cell wall matrix, and the molecular makeup of its polysaccharides. Differences at each of these levels could influence where and when fermentation may happen in the colon, regardless of the total dietary fibre content of barley [5,6].

The duration and timing of short-chain fatty acid (SCFA) exposure are particularly important because these chemicals affect gut hormones and neural pathways involved in appetite and energy balance [7]. Previous reviews have examined how food structure, particularly that of wheat and highland barley, affects microbial use of carbohydrates [8,9,10], have described SCFA production pathways and how they interact with host receptors, and have linked their production to disease [11]. In addition, a recent framework proposes that both the timing of fermentation and the gut transit rate may influence where fermentation occurs along the colon and may therefore affect host physiology [6,12,13]. This is an exciting concept, but to date, researchers have used isolated fibres and have not focused on grain morphology or its effects on gut hormones and host signalling.

## 2. Search Strategy

We carried out a narrative review of the literature using PubMed, Scopus, and Google Scholar. We grouped search items into three main themes based on the review’s conceptual pathway: 1. Barley structure and the impact of processing (i.e., barley β-glucan, resistant starch (RS), grain morphology, cell wall encapsulation, milling, gelatinisation); 2. Microbial fermentation (i.e., gut microbiota, colonic fermentation, SCFAs, cross-feeding, fermentation kinetics, colonic transit time); and 3. Host signalling (i.e., GLP-1, PYY, INSL5, enteroendocrine cells, L-cells, FFAR2, FFAR3, satiety, appetite regulation). We used Boolean operators to combine terms from these groups. For example (“barley” OR “β-glucan” OR “resistant starch”) AND (“colonic fermentation” OR “SCFA”) AND (“GLP-1” OR “enteroendocrine”).

We focused on peer-reviewed primary research articles and systematic reviews published between 2010 and March 2026. We also included earlier foundational work if it offered important mechanistic insights not found in more recent studies. This review covers human intervention trials, animal models, and in vitro fermentation studies to inform mechanistic frameworks. We focused on whole-grain and minimally processed grain matrices, using barley as the main model.

We excluded studies that used isolated or synthetic fibres in the structural and processing sections but retained mechanistic studies using purified substrates when they provided evidence of microbial utilisation pathways. We also excluded studies not published in English. Since the studies employed various experimental models and barley preparations, a formal meta-analysis was not possible. Instead, we used a narrative synthesis to combine mechanistic evidence from structural, microbiological, and physiological research, highlighting knowledge gaps that informed the testable hypotheses in Section 8.

## 3. Barley Grain Structure: Fermentable Substrates

**Figure 1:**
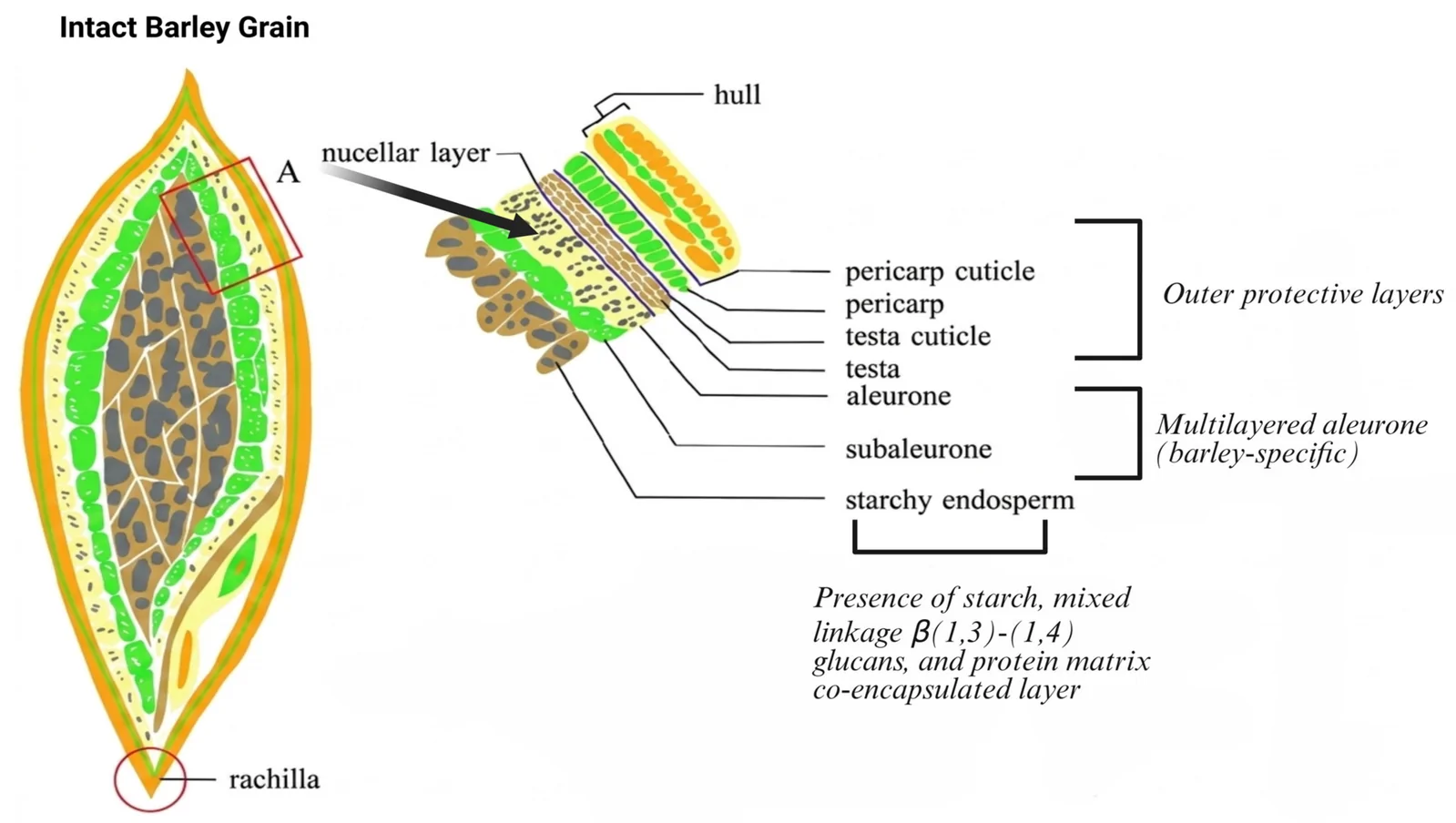
Diagram of the longitudinal section of a mature barley grain shows a layered structure from the outer hull to the starchy endosperm. Glucose accounts for 75–79% *w*/*w* of starchy endosperm wall monosaccharides, and arabinose plus xylose accounts for 19–22% *w*/*w* [16,17]. The inset (panel A) highlights tissue layers involved in carbohydrate encapsulation: the outer protective layers (pericarp cuticle, pericarp, testa cuticle, testa), the multilayered aleurone (thicker in barley), the sub-aleurone, and the starchy endosperm with starch granules enclosed by cell walls and embedded within a hordein protein matrix. This structure supports the carbohydrate-encapsulation system described in Section 3. Adapted from [18] DOI: 10.1093/fqsafe/fyac012. Licensed under Creative Commons Attribution 4.0 International (CC BY 4.0). Edited in BioRender. Chakraborty, S. (2026) https://BioRender.com/jpmhgnm.

Barley is a useful model for studying how different levels of structure (Figure 1) affect carbohydrate accessibility in grains and when they become available for microbial fermentation in the colon. The starchy endosperm makes up about 77 to 82% of the grain’s dry mass (DM) and is the main region for storing starch (51–65% DM) and β-glucan (3.7–6.5% DM). β-glucan accounts for about 75% of the endosperm wall [14]. The outer layers, namely, the bran, pericarp, and aleurone, make up about 10–15% of the grain’s mass and are rich in non-starch polysaccharides. The cell walls of the aleurone layer are dominated by arabinoxylan: monosaccharide analysis of isolated barley aleurone walls gives arabinose plus xylose at 67% *w*/*w* and glucose at 29% *w*/*w*, of which 91% is mixed-linkage β-glucan, which is the inverse of the starchy endosperm wall [15]. These structural differences act as barriers that limit how readily digestive enzymes and gut microbes can access carbohydrates, which may then influence when and where fermentation occurs in the colon (the influence on location and timing is discussed in detail in Section 5 and Section 6).

### 3.1. Starch and Resistant Starch Organisation

In barley endosperm, starch is mainly stored as amylose, which is mostly linear with some branch points, and amylopectin, which is branched with many short chains [17,19]. Barley grains with 25–30% amylose are considered normal, although the less common, waxy types of barley have less than 5% amylose. Since amylopectin has short branches, it gelatinises faster and more completely than the longer, more linear amylose chains [20,21]. The amount of amylose and the structure of starch granules affect which type of resistant starch (RS) remains in the colon following digestion [22]. High-amylose barley has more than 35% RS [19,20,23]. Resistant starch is currently divided into five types, numbered from 1 to 5: resistant starch type 1 (RS1) is trapped within intact cell walls, which slow enzyme access, and is common in coarsely milled or whole barley; resistant starch type 2 (RS2) is an ungelatinized starch with a tightly packed structure that resists amylase and is found in raw or minimally processed barley; resistant starch type 3 (RS3) forms when gelatinised starch cools and sets; resistant starch type 4 (RS4) is made by chemical modification and does not occur naturally in barley; resistant starch type 5 (RS5) consists of amylose-lipid complexes formed during heating with lipids, and it adds a small amount of RS to processed barley. Although a recent review has expanded the classification of resistant starch (RS) to 10 types, depending on preparation and/or formation [24], the five-type classification system remains standard in the literature. In minimally processed barley, RS1 and RS2 are most common because cell walls and the crystalline structure of granules are the main barriers [22,24]. However, the actual ratio of these starch types in barley does not match the classification labels across the literature. Isolated native highland barley starch contains only around 3% resistant starch (RS2) of total starch [25], making RS2 a minor component. Consequently, most resistant starch reaching the colon from whole or coarsely processed barley is likely RS1, characterised by preserved cell wall structure rather than specific chemical properties. The fermentable carbohydrates in barley vary in structural resistance, position within the grain, response to processing (Table 1), and how well their behaviour in the colon has been studied.

**Table 1:** Major fermentable carbohydrates in barley: structural basis of resistance, distribution within the grain, and responses to processing.

Major Barley Carbohydrates	Basis of Resistance	Distribution in Grain	Response to Processing	References
Mixed-linkage β-glucan	(1,3;1,4)-β-D-glucan; no human β-glucanase. Viscosity limits diffusion; forms a hydrogen-bonded network with starch that restricts enzyme access independently of retrogradation (in vitro, isolated highland barley starch).	75–79% *w*/*w* of starchy endosperm wall monosaccharides; 29% *w*/*w* of aleurone wall, of which 91% is 1,3;1,4-β-glucan. Whole grain 4.3–8.9% dw; 2.0–4.0% in the outer 10%, peaking in the endosperm at 30–60% removal in 7 of 9 hull-less cultivars.	Pearling concentrates β-glucan in the residual kernel. Milling and heat reduce its molecular weight and solubilises it; extrusion depolymerises it. Baking or pasta processing do not significantly alter β-glucan and phenolics, and β-glucan is enriched in post-digestion residue (in vitro, wheat–barley blended foods).	[14,15,26,27,28]
Arabinoxylan	β-(1,4)-xylan backbone with arabinofuranosyl substitution at O-2 and/or O-3. Barley aleurone is more substituted than wheat (Ara:Xyl 0.52 vs. 0.35), with O-2 substitution in barley, while wheat is mainly O-3 substituted.	Ara + Xyl 19–22% *w*/*w* of starchy endosperm wall monosaccharides; 67% *w*/*w* of aleurone wall. Decreases from outer to inner pearled fractions, correlated with bound ferulic and *p*-coumaric acid.	Removed by pearling, tracking bound phenolic acid. Ester-linked phenolic acids total 1.2% of the barley aleurone wall, below wheat at 1.8%.	[14,15,29]
RS1 (physically inaccessible)	Starch is physically entrapped by intact cell walls, which act as a barrier rather than the starch itself. Amylase-sized fluorescent probes barely penetrate raw or cooked highland barley endosperm cell walls.	Starchy endosperm. Not quantified separately from RS2 in barley in any retrieved study.	Chyme-retaining kernel structure was less digestible than flour across five highland barley varieties (in vitro). Isolated intact cells retained 61.80% starch digestibility after 6 h at 80 °C, compared to 83.35% in crushed flour; enzymatic wall degradation made the digestion rate similar (0.0095 vs. 0.0099 min^−1^).	[30,31,32]
RS2 (native granular)	Native granule crystallinity resists α-amylase; resistance increases with amylose content.	Endosperm amyloplasts. Hull-less cultivars span 0–7% amylose (waxy), ~25% (normal) and 36–42% (high amylose); waxy and high-amylose lines carry more β-glucan than normal-starch lines.	Abolished by gelatinisation. In highland barley starch (amylose 23.5%), native resistant starch is 3.27%. Heat–moisture treatment at 110 °C for 4 h increased resistant starch to 9.77% (20% moisture), 12.52% (25%), and 9.35% (30%). Dry heat at same temperature reduced it to 1.84–2.21%, increasing slowly digestible starch. Endogenous protein and β-glucan form a matrix on the granule surface that inhibits digestion (in vitro, isolated starch).	[25,27,33]
RS3 (retrograded)	Recrystallised double helices of retrograded amylose.	Not native to the grain; formed during processing of barley foods.	Cook–cool cycling drives it. Added barley β-glucan delays retrogradation through steric hindrance and competition for water while raising viscosity via a starch–β-glucan network (in vitro, isolated starch). Extrusion alters starch–β-glucan structure and digestive resistance (in vitro).	[26,34]

Note: the model system is stated in parentheses for each experimental finding; The distribution column reports monosaccharide compositions of isolated cell wall preparations on a % *w*/*w* basis; RS4 is produced by chemical modification and does not occur in barley. RS5 is formed in trace amounts during thermal processing and has not been characterised in barley fermentation. Abbreviations. Ara, arabinoxylan; RS, resistant starch; DW, dry weight.

### 3.2. β-Glucan and Arabinoxylan

β-glucans are non-starch polysaccharides located in the cell walls surrounding the starch-containing endosperm cells of barley, where they are the dominant polysaccharide [14]. Barley’s endosperm cells are different from those in other cereals because they have thicker cell walls and a unique multi-layered aleurone. This is why barley keeps its fibrous parts unless it is very finely processed [35]. In barley, β-glucans have 2–3 β-(1,4) linkages interrupted by a β-(1,3) linkage, which creates alternating cellotriose (DP3; Degree of Polymerisation 3) and cellotetraose (DP4) units throughout the polymer. The ratio of DP3 to DP4 units and the overall molecular size differ among barley species, leading to changes in physicochemical properties and physiological effects [36]. The polysaccharide-utilisation loci (PUL) are present in certain bacterial species and strains and are essential not only for breaking down β-glucan but also for supporting the growth of other essential microbes through cross-feeding. For example, the surface-binding protein for *Bacteroides ovatus* (BoSGBPMLG-A) and *Segatella copri* (*Prevotellaceae*) is characterised by MLG-PUL with a GH16 endo-glucanase [37,38]. Additionally, a synthetic ecology study using batch and continuous culture showed that *Bacteroides ovatus* extracellularly depolymerised barley β-glucan, releasing DP3 and DP4 oligosaccharides and supporting the growth of *Subdoligranulum variabile* and *Hungatella hathewayi* (formerly *Clostridium hathewayi*), confirming a cross-feeding relationship [39]. β-glucan may affect starch digestibility not only through encapsulation but also via direct molecular interactions. When barley β-glucan is added to barley starch, it delays retrogradation by creating steric hindrance and competing for water, while forming a hydrogen-bonded β-glucan–starch network that increases viscosity independently of β-glucan’s own viscosity [26]. This network limits enzyme access without relying on starch recrystallisation, representing a distinct mechanism from cell wall encapsulation and bulk viscosity. It functions whenever β-glucan and starch are in molecular contact, even in disrupted matrices where the wall barrier is absent. Arabinoxylan, the other major cell-wall polysaccharide, is distributed inversely to β-glucan: it is minor in the starchy endosperm wall but the dominant constituent of the aleurone wall [17,35,40]. Its content also decreases from the outer to the inner pearled fractions of the grain, correlated with bound ferulic and *p*-coumaric acid [29].

### 3.3. Barley Endosperm: Co-Encapsulation Layer

**Figure 2:**
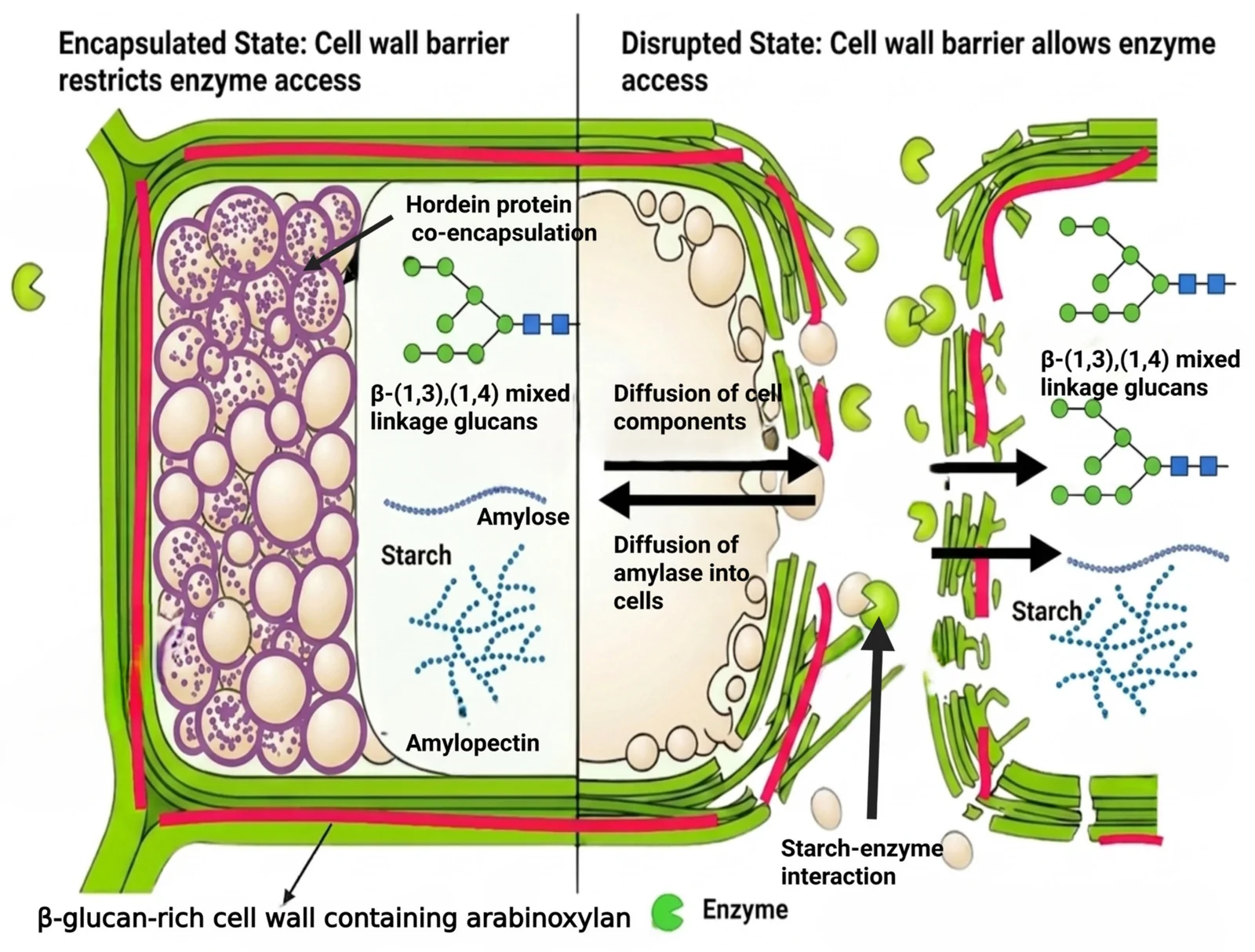
This illustration shows how starch is stored within barley endosperm and how breaking cell walls influences enzyme access. The left panel features an intact endosperm cell, where starch granules are shielded by a β-glucan-rich cell wall containing arabinoxylan that restricts enzyme entry, along with a hordein protein coating, forming a co-encapsulation system. Inside the cell, starch containing amylose and amylopectin is present; β-glucan is a constituent of the surrounding wall. Amylase outside the cell cannot penetrate the cell wall. The right panel illustrates a structurally disrupted cell in which mechanical processing has compromised cell-wall integrity, allowing enzymes greater access to intracellular starch. Thermal processing can alter starch and protein organisation but does not necessarily abolish an intact cell-wall barrier. Enzymes can then directly interact with starch at the boundary. The extent of residual co-encapsulation influences starch degradation and the release of fermentable substrates. Created in BioRender. Chakraborty, S. (2026) https://BioRender.com/juwcfvi.

The cell wall is porous, and the pore size largely limits amylase access to the cell interior. The internal protein matrix, which forms a composite with the cell wall, also limits amylase access (Figure 2). Cooking does not always abolish this barrier. A study showed that amylase-sized probes fail to penetrate the walls of isolated highland barley endosperm cells whether raw or cooked, and progressive enzymatic degradation of the wall raises starch digestibility toward that of mechanically crushed flour [31]. Encapsulation is therefore lost by mechanical disruption rather than by thermal treatment alone. Another foundational study identified a protein matrix in barley grains composed of hordeins (major endosperm proteins) that surrounds the starch granules and is interconnected throughout the endosperm [41]. Because the cell wall and the protein matrix are not separate layers but rather a composite system, the cell wall limits enzyme entry at the outer surface, while the protein matrix slows amylase at the granule surface [41]. Hordeins are the main storage proteins, making up 30–50%, while glutelin accounts for 35–45% of the total barley storage protein fraction. Protein Z is the major barley albumin protein [42]. More recently, a study showed that, upon cooking highland barley, scanning electron microscopy revealed a continuous protein matrix encapsulating the starch granules, which may have slowed access to amylases [43]. Additionally, another study by Xie et al. (2024) showed that after removing non-starch components, such as β-glucan and proteins, from highland barley, the estimated glycaemic index (GI) increased and resistant starch content was significantly reduced [33].

## 4. Effects of Processing on Grain Structure

Techniques such as milling, heating, fractionation and de-branning alter the chemical makeup of barley, as well as the structure, distribution, and organisation of its polysaccharides [36,44].

### 4.1. Mechanical Size Reduction

**Figure 3:**
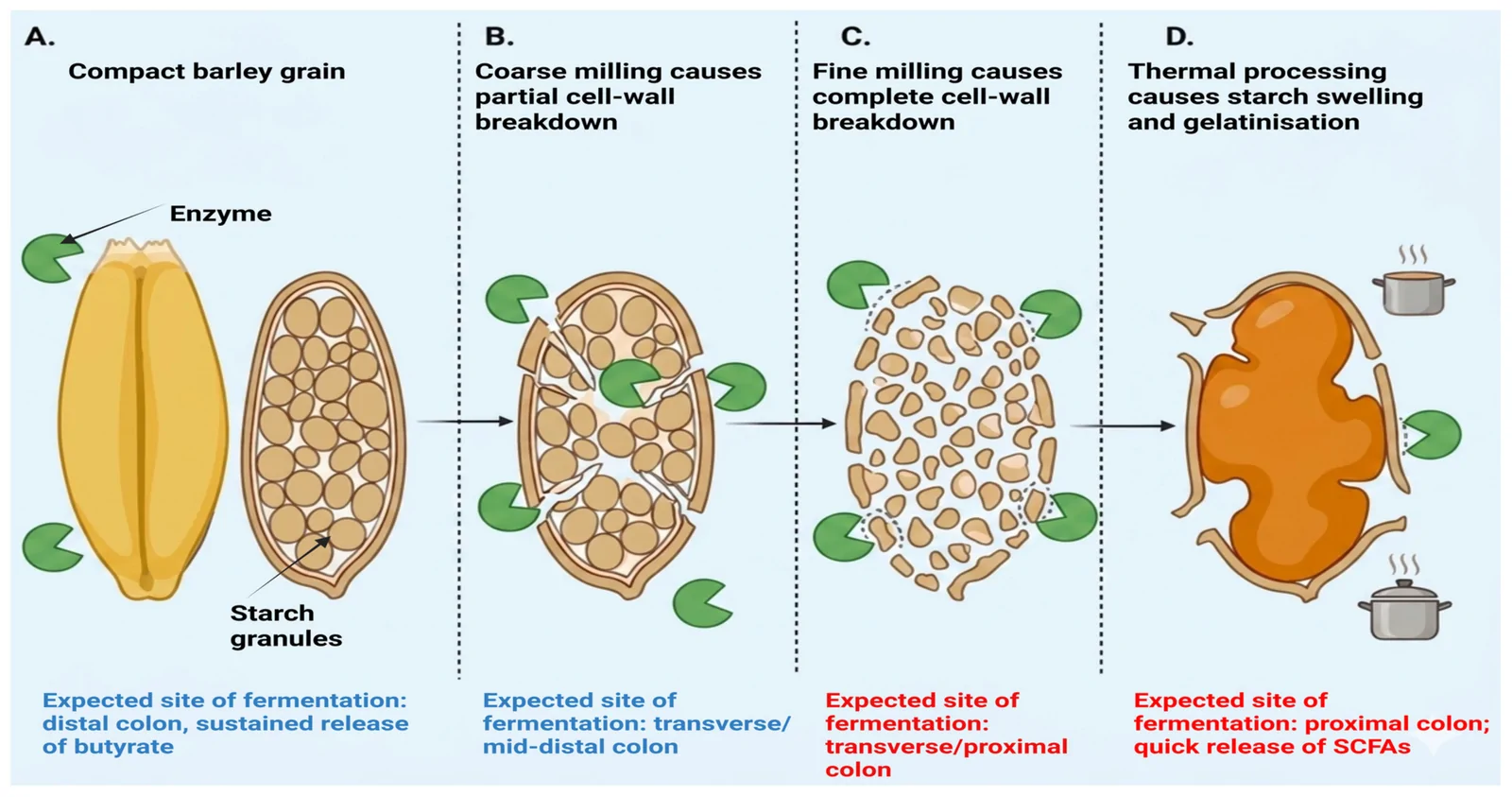
Progressive processing reduces barriers to enzyme access in barley endosperm. (**A**) Native barley has a dense matrix with starch inside intact cell walls and proteins, limiting enzyme reach. (**B**) Coarse milling partially disrupts cell walls, exposing some starch, but fragments and proteins hinder access. (**C**) Fine milling nearly removes wall encapsulation, increasing starch surface and enzyme accessibility. (**D**) Thermal treatment gelatinises starch within already-disrupted cells; the cell wall itself remains a barrier to enzyme entry. The progression shows increased carbohydrate accessibility, possibly shifting fermentation from the distal to the proximal colon. Figure by Microsoft^®^ PowerPoint and BioRender. Chakraborty, S. (2026) https://BioRender.com/w4m78vi.

Recent studies show that kibbling and fine milling can reduce wheat particle size and break cell walls, exposing starch and non-starch polysaccharides that were previously protected [9]. This allows digestive enzymes in the upper gastrointestinal tract to access these molecules [8,45] (Figure 3). Recently, an in vitro study reported that larger or more intact grain particles slow enzyme access and delay the onset of fermentation [32], suggesting that structural barriers may be better preserved. Another study on highland barley directly showed the contrast in fermentation output. Milling intact barley kernels into flour increased starch digestibility from 29% to 76%. The higher starch digestibility was accompanied by higher early fermentation activity, but lower total short-chain fatty acid yield over 48 h in an in vitro model [10]. The greater early fermentation activity observed after intensive milling is consistent with more rapid substrate utilisation and could, in vivo, favour earlier fermentation. These findings are consistent across the literature available to date; however, the specific structural regions of the grains affected by coarse versus fine milling remain largely unknown. Therefore, until targeted, coupled imaging and fermentation studies fully characterise these structural changes, the true effect of grain processing on fermentation will remain unclear.

### 4.2. Thermal and Thermochemical Treatments

Heating grains through steaming, boiling, or roasting alters barley starch structures by breaking bonds within the grain matrix, causing starch swelling, loss of granular structure, and birefringence. This process is known as gelatinisation [46]. These modifications decrease the resistant starch fraction because gelatinised starch is more easily hydrolysed once enzymes have access to it. However, gelatinisation does not break open the cell wall (Section 3.3), so in intact structures, the enhanced accessibility mainly affects cells with already disrupted walls [47]. Because β-glucan dissolves under the same hydrothermal conditions that gelatinise starch, cooking brings the two polymers into molecular contact. Thus, processing forms interactions between solubilised β-glucan and retrograding amylose rather than breaking them apart, unlike an encapsulation barrier. Additionally, when starch cools after heating, retrogradation occurs, leading to RS3 formation, which enables more material to reach the colon and may slow fermentation [48]. A study shows that sand-roasted highland barley, rather than flaked or milled, retained more of its structure [10]. These studies suggest that conditions such as cell wall breakdown, gelatinisation, and retrogradation may affect how much starch escapes upper-gut digestion and how accessible it is to enzymes. These changes may influence the amount and rate at which fermentable material becomes available to the colonic microbiota and could therefore affect the timing, and potentially the regional distribution, of fermentation.

### 4.3. Fractionation and De-Branning

Fractionation (isolation of grain macromolecules) and de-branning (removing the bran layer from the grain), also termed pearling, redistribute grain components anatomically. For example, β-glucan content can vary by genotype and growing environment, and dry fractionation can concentrate β-glucan into particular milling streams [49,50]. Additionally, pearling sequentially strips away the outer layers of the kernel, which in hull-less barley contain the lowest levels of β-glucan. Specifically, β-glucan makes up 2–4% of the dry weight in the outermost 10% (pericarp and testa) and approximately doubles in the next 10% (aleurone). It peaks in the endosperm after 30–60% of the kernel is removed in most genotypes [27]. Consequently, pearling mainly removes outer material rich in arabinoxylan and phenolics [29], while the β-glucan content becomes concentrated in the remaining kernel.

## 5. Colonic Microbial Ecology and the Influence of Cross-Feeding on Colonic Fermentation

How starch and non-starch polysaccharides that reach the colon are processed and digested (as discussed in Section 3 and Section 4) may influence which bacterial species can utilise the nutrients and how quickly. In addition, different types of non-starch polysaccharides reaching the colon have distinct effects on the bacterial community and its activity [51]. In this context, both the microbes present in the colon (i.e., microbial ecology) and their dependence on each other for substrates (i.e., cross-feeding) likely shape the products of fermentation of processed grains and their downstream metabolic effects. Colonic transit time also likely modifies these outcomes.

It is worth noting that, due to substantial recent changes in bacterial taxonomic nomenclature, bacterial names were checked against the List of Prokaryotic names with Standing in Nomenclature (LPSN) and recent taxonomic literature [52]. Where a name has been revised since the cited source was published, the name used in that source is provided in parentheses at first mention (e.g., ‘*Bacteroidota*’ formerly ‘*Bacteroidetes*’) [53]. Names that are unchanged remain without annotation. This aligns the review with current taxonomy while preserving its traceability to the primary literature.

The widely used approach of 16S rRNA gene amplicon sequencing reveals bacterial community patterns but cannot identify the specific pathways organisms use. This requires evidence such as growth on defined substrates, enzyme- or gene-level data, or characterisation of polysaccharide utilisation loci (PULs). For this reason, organisms identified through abundance shifts are best termed as candidate responders rather than confirmed degraders [54,55].

The colonic microbiota operates across trophic levels, with primary degraders (responders) constituting the first tier. These organisms initiate fermentation by attacking intact barley non-starch polysaccharides. Several taxa have been implicated in the initial or downstream utilisation of barley-derived carbohydrates. For example, *Ruminococcus bromii* is a well-known resistant starch degrader [56], while members of *Bacteroides* spp. are known to possess substrate-specific PULs [57,58]. *Blautia* spp. may contribute through downstream acetogenesis. Resistant starch and cereal β-glucans are metabolised through substrate-specific trophic networks rather than by just a single organism, because the gut microbiota rely on specialised carbohydrate-active enzymes (CAZymes) and PULs to sense, bind, depolymerise, and import distinct dietary glycans [38,59,60]. For example, *Bacteroides ovatus* prefers plant glycans with structural complexity and hemicellulose-targeting loci, whereas *Bacteroides thetaiotaomicron* relies more extensively on host mucin glycan utilisation [61,62].

Mixed-linkage β-glucan utilisation is now mechanistically resolved in several organisms, including *Bacteroides ovatus* and related organisms that carry specific utilisation loci. *Segatella copri* (formerly *Prevotella copri*) [63] appears to extend the primary degradative capacity beyond *Bacteroides* through a distinct *Prevotellaceae* system and is therefore assigned to Tier 1 rather than to secondary fermenters. Protein-level specificity, such as differences in surface-binding proteins and glycoside hydrolases, shapes the β-glucan utilisation capability of organisms [38,64]. Primary degraders (responders) have also been reported to produce short-chain fatty acids such as acetate, propionate, and succinate, whereas butyrate production is negligible. The secondary fermenters in Tier 2 include *Bifidobacterium* and lactobacilli (previously all grouped within *Lactobacillus*) [65]. These organisms then consume the oligosaccharides broken down by Tier 1 members, often producing an overlapping yet distinct metabolite profile, including acetate, propionate, and lactate [32,66]. Lactate is suggested to be the characteristic Tier 2 output, especially for *Bifidobacterium* (via the bifid shunt, distinct phosphoketolase pathway) and lactobacilli. Tier 3 species are primarily butyrate producers, including *Faecalibacterium prausnitzii*, *Roseburia intestinalis*, *Megasphaera indica*, and *Anaerobutyricum hallii* (formerly *Eubacterium hallii*) [67]. These taxa are commonly associated with butyrate production and often utilise fermentation intermediates such as lactate and acetate, although substrate-use capacity varies among species and strains [68,69]. The microbial network can therefore be conceptualised as a sequential cross-feeding cascade, while recognising that functional overlap occurs among taxa. Some lineages historically reported within *Faecalibacterium prausnitzii* have subsequently been resolved as distinct species, including *F. duncaniae*, *F. hattorii*, and *F. gallinarum*. Species-level attribution in older butyrate studies should therefore be interpreted with caution [70].

Cross-feeding is a key mechanism in gut ecology. Primary β-glucan degradation releases oligosaccharides, sustaining other organisms along the cross-feeding chain [39]. Similar ecological studies on resistant starch show that only a limited set of organisms can initiate their breakdown, but butyrate production depends on interactions between degraders and secondary fermenters rather than on degraders alone [60,71]. Community output is also shaped by glycan system structure [72].

Fermentation outcomes in both human and in vitro fibre studies have been shown to vary based on substrate structure and the baseline microbiome composition. Studies show that fibre breakdown does not reliably translate into higher butyrate unless the appropriate primary degraders and cross-feeding partners are already present [71,73]. Overall, these findings indicate that the physical state of barley-derived substrates in the colon, whether as intact polymers, oligosaccharides, or granular resistant starch, may influence which microbial groups are involved and the specific short-chain fatty acid profile produced. This is consistent with the architectural hypothesis proposed in this review.

### Influence of Colonic Transit Time on Fermentation Outcomes

Colonic transit time and substrate delivery may modulate the site of colonic fermentation. Non-digestible polysaccharides enter the caecum and proximal colon first, and when fermentable carbohydrates are depleted, microbial metabolism can shift from saccharolysis to proteolysis [74]. Human marker studies show that longer colonic transit is associated with overall microbiota composition, higher richness, and a metabolic shift toward protein catabolism, indicating that transit time is a key factor for both community structure and function [75].

Distal fermentation has attracted particular attention because butyrate generated in the distal colon is believed to enhance epithelial integrity and provide other colonic benefits, which is crucial since colorectal disease frequently develops in this area [76,77]. Additionally, the presence of substrate reaching this far suggests continuous rather than rapid utilisation. Rat studies indicate that BARLEYmax, containing fructan, β-glucan, and resistant starch, boosts distal colonic acetate and overall short-chain fatty acids, unlike a high β-glucan variety [3]. This rat-based study is the only barley study measuring regional fermentation; the human barley interventions mentioned here did not assess regional fermentation, so caution must be exercised when extrapolating (Table 2). This limitation matters because faecal short-chain fatty acids are only a small fraction of total colonic production [78] and can correlate inversely with transit time, making stool endpoints alone an imperfect readout of fermentation location or magnitude [75,79]. β-glucan is rapidly depleted in vitro and may therefore be butyrogenic but ferments too quickly to reach the distal colon. Butyrate yield and the site of butyrate production are distinct properties, and neither can be inferred from the other. In addition, although evidence supports the idea that a structurally preserved grain matrix may sustain all three tiers sequentially and deliver substrate progressively, evidence remains limited [71] on how structurally distinct grain matrices might control the rate and sequence of metabolite transfer among the three tiers.

Whether dietary means can shift fermentation distally, and whether doing so is beneficial, remains unclear. In a 12-week human trial, an isolated mixed-fibre supplement designed to increase distal fermentation produced increased colonic permeability and circulating interleukin-6 with no change in insulin sensitivity [80]. Additionally, increased delivery to the colon is not uniformly beneficial, and when relevant primary degraders or cross-feeding partners are absent, gas production may increase without a corresponding increase in butyrate [71,73], thereby imposing a symptom burden in individuals with impaired gastrointestinal homeostasis. It has not yet been tested whether a grain matrix that maintains its structure and releases substrate gradually, rather than as a bolus of isolated fibre, sustains fermentation without these effects.

**Table 2:** Evidence base for the fermentation and host-signalling claims discussed in this review, classified by substrate and model system.

Main Findings	Substrate	Model System	Relevance to the Present Review	Limitations	References
1. Both barley diets increased caecal acetate, butyrate, and total SCFA (53.6 vs. 27.3 µmol/g in control). BARLEYmax raised acetate and total SCFA in the distal colon, despite having less β-glucan (6.3 vs. 9.0 g/100 g) but more fructan (9.0 vs. 2.2) and resistant starch (3.0 vs. 0.5 g/100 g). Barley increased *Bacteroidota* and decreased *Bacillota*.	Whole barley lines in the diet; cellulose control	Rat, 4 weeks; caecal, proximal and distal colonic digesta sampled	Mixture of carbohydrates fermenting at different rates sustains fermentation further into the distal colon than high β-glucan alone	Rat model; not investigated in humans. The two barley lines differ in three carbohydrates simultaneously, so no single component can be isolated.	[3]
2. Barley kernel bread improved postprandial glucose and insulin, with marked inter-individual variation. Responders showed a *Segatella copri* bloom and enrichment of β-glucan and complex-polysaccharide degradation genes; *S. copri* (formerly *Prevotella copri*) improved glucose tolerance in mice only when dietary fibre was available.	Barley kernel bread vs. white wheat bread	Human crossover, with shotgun metagenomics and mechanistic mouse experiments	Metabolic response to intact barley is microbiota-dependent, and functional microbiota differences need not appear in faecal SCFA	No SCFA profile attributable to barley and no fermentation-site data. No organism shown to degrade barley.	[81]
3. β-glucan, RS and phenolics survived upper-GI digestion; β-glucan was rapidly fermented, with most depletion within 10 h and near-complete by 24–48 h. Fermentation was strongly butyrogenic: butyrate 36–43% of total SCFA, highest in the high-β-glucan genotypes. Genotypes converged toward similar communities by 24–48 h, while metabolic outputs differed more than final taxonomy.	Extruded whole barley, four hull-less genotypes	In vitro: extrusion → INFOGEST digestion → pooled human faecal fermentation	An intact whole-grain barley matrix survives digestion and gives a butyrogenic profile, unlike isolated β-glucan	No human data; no regional resolution. Genotypes differ in β-glucan, starch type and phenolics simultaneously. Rapid β-glucan depletion is consistent with relatively early substrate utilisation; however, this batch-fermentation model cannot determine proximal versus distal colonic fermentation.	[82]
4. Fermentation of cereal β-glucans gave acetate: propionate: butyrate (51:32:17) at 24 h; 172 kDa barley fraction reached 37.1% propionate at 48 h.	Isolated β-glucans, barley and oat pooled	In vitro pH-controlled batch fermentation, human faecal inoculum, *n* = 3 donors	β-glucan fermented in isolation is propionate-rich rather than butyrogenic	Not barley-specific, as barley and oat values were pooled. No grain matrix; three donors only.	[83]
5. β-glucan was enriched in the post-digestion residue and fell to <0.30 g/100 g DW by 48 h of fermentation, with increases in acetate and propionate.	Crackers, cookies and fresh pasta made from combined wheat and barley flour	In vitro gastrointestinal digestion followed by colonic fermentation	β-glucan survives upper-GI digestion in a real food matrix and is subsequently fermented	Not barley-only, so the profile cannot be attributed to barley alone.	[28]
6. Acetate infused into the distal colon altered circulating metabolic markers and host signalling.	Sodium acetate	Human, direct colonic infusion	SCFAs delivered distally produce measurable host responses	No barley and no food matrix; a single infused acid rather than endogenous fermentation.	[12]

Note: No human study has measured the SCFA profile or the anatomical site of fermentation of a structurally defined barley preparation. The only regional measurement in barley is from a rat study [3]. The only human barley intervention with microbiome profiling measured glycaemic endpoints with no difference in faecal SCFAs between responders and non-responders [81], and the only human regional SCFA data derive from infusion of a single acid rather than from fermentation of a food [12]. Comparisons between barley genotypes are confounded by variations between β-glucan content and starch type.

## 6. Microbial Metabolites and Host Signalling

**Figure 4:**
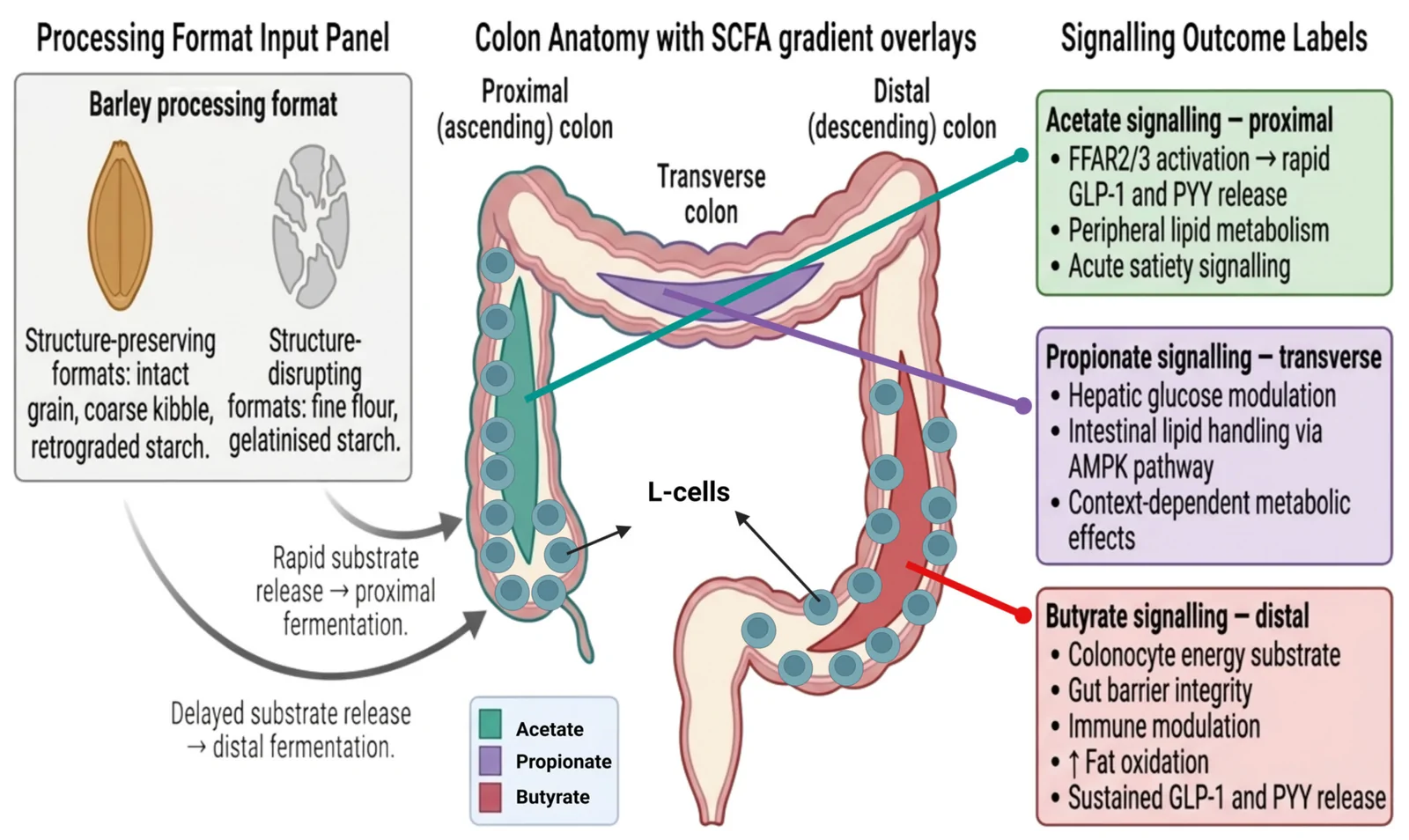
Proposed conceptual framework linking barley grain-matrix integrity with colonic fermentation dynamics and regional host signalling. The chosen processing method is proposed to influence where fermentation occurs along the colon and its rate of release [91,92]. This may influence regional SCFA profiles and host responses. Structure-preserving formats, such as coarsely kibbled grain, may slow substrate release, directing fermentation towards the distal colon [10,32], whereas disrupting formats, such as fine flour, may encourage fermentation in the proximal region [9,45]. Short-chain fatty acid products (acetate, propionate, butyrate) may be produced in specific areas depending on substrate availability and microbial presence [93,94]. Subsequent regional differences in enteroendocrine L-cell population density and FFAR2/3-receptor-mediated signalling suggest that the fermentation site plays a key role in engaging these metabolic pathways [13,86]. Created in BioRender. Chakraborty, S. (2026) https://BioRender.com/1l1zakt.

The role of short-chain fatty acids may depend not only on how much of them is produced, but also on where, when, and for how long they are produced in the colon. The types of receptors and the location of hormone-producing cells vary along the colon, which affects the signals triggered [84,85,86]. When short-chain fatty acids are present, they activate free fatty acid receptors (FFAR2 and FFAR3) on L-cells lining the colon, leading to the release of hormones such as glucagon-like peptide-1 (GLP-1) and peptide YY (PYY), depending on the amount and location of the receptors [87,88]. These receptors are more common in the distal small intestine and colon, where L-cells are more abundant. In the colon, L-cells release GLP-1, PYY and INSL5 when short-chain fatty acids activate receptors such as FFAR2, FFAR3, and TGR5 [86,89]. GLP-1 and PYY may reduce appetite and slow colonic motility, while INSL5, expressed only in colonic L-cells, may increase hunger and glucose production in the liver. This suggests that colonic L-cells act as energy sensors, connecting fermentation signals to the host’s metabolism [89,90]. As a result, the native barley structure may not only influence the amount of short-chain fatty acids produced, but also where they appear in the colon and which L-cells are involved (Figure 4). So far, no human study has directly examined these signalling pathways using structurally preserved versus disrupted grain matrices.

### Gut–Brain Axis and Satiety

The differences in GLP-1 and PYY release from L-cells in the proximal and distal colon depend more on what these cells do than just on where they are located. Studies in mice show that L-cells in the distal colon produce about twice as much GLP-1 per cell as those in the upper colon, and that they release PYY and INSL5 when stimulated by short-chain fatty acids, triggering a long-lasting, complex hormonal response [85,86]. Experiments in genetically modified mice treated with a drug that activates lower-colon L-cells show that these cells can function as an endocrine organ. Activating these cells improves postprandial glucose via GLP-1 and reduces food intake via PYY, even without the usual GLP-1 receptor [89]. Consistent with this in humans, a study showed that while distal colonic acetate infusion in overweight adults increased fasting fat oxidation and PYY levels, proximal colonic infusion produced no significant effects, suggesting that fermentation site may play a key role in governing host signalling [12]. The types and amounts of SCFAs are likely to affect L-cell signalling. Acetate and propionate are the strongest activators of L-cells via FFAR2. Whether barley fermentation in the distal colon favours butyrate over acetate and propionate remains unclear, including whether available profiles differ by substrate form. Additionally, it is unclear whether butyrate directly activates L-cells via FFAR2 or FFAR3, or whether it primarily acts on the cells lining the colon where it is produced, with only indirect effects on hormones [86,87]. These regional differences in L-cell density and function provide a plausible basis for linking the site of fermentation to host hormonal signalling, but no human study has yet explored this area with structurally defined barley preparations; the requirements for doing so are discussed in Section 8.

## 7. Negative Effects of Ultra-Processing

Ultra-processing of grains such as barley dismantles much of the grain’s natural carbohydrate architecture. The progression from intact grain to refined, extruded, or otherwise highly processed products disrupts cell-wall structures, reduces native starch granule organisation, lowers starch crystallinity, may depolymerise β-glucan, and diminishes viscosity. Importantly, the most significant consequence of ultra-processing is often not a fundamental change in carbohydrate chemistry itself, but rather the alteration of the grain’s multiscale physical structure, which has evolved to regulate digestion, nutrient accessibility, and the rate of nutrient release [95,96].

Diets rich in highly processed carbohydrates and ultra-processed foods may adversely affect the gut ecosystem by providing rapidly digestible carbohydrates while supplying relatively little microbiota-accessible fibre. This dietary pattern can reduce microbial diversity, decrease the abundance of health-associated bacteria such as *Faecalibacterium prausnitzii*, and lower SCFA production, particularly butyrate, which plays an important role in maintaining intestinal barrier integrity and regulating immune and inflammatory responses [97,98]. These changes may impair colonisation resistance against opportunistic microorganisms, promote intestinal barrier dysfunction, and contribute to chronic low-grade inflammation [99].

Although evidence directly linking processed carbohydrates to intestinal protozoan parasite infections remains limited, diet-induced dysbiosis may influence host immunity and microbial interactions that affect susceptibility to both bacterial and parasitic pathogens. Consequently, individuals with disturbances in gastrointestinal homeostasis, including irritable bowel syndrome, inflammatory bowel disease, post-infectious dysbiosis, or other conditions associated with impaired gut-barrier function, may be particularly susceptible to symptom exacerbation and further microbiome disruption when consuming diets high in highly processed carbohydrates and ultra-processed foods [99,100].

## 8. Conclusions

Current evidence suggests that barley grain structure affects carbohydrate accessibility during digestion and can influence fermentation kinetics. Factors such as cell-wall encapsulation, resistant starch organisation, and the physical form of β-glucan may explain how processing changes the substrates reaching the colonic microbiota. However, no human research has directly examined the specific site of fermentation or analysed regional SCFA profiles using structurally defined barley preparations. Consequently, it remains unclear whether maintaining the barley matrix shifts fermentation to more distal parts of the colon or produces different regional metabolite profiles. We propose that barley formats which preserve structure may prolong substrate availability and potentially extend fermentation further along the colon, while highly disrupted formats may promote faster fermentation. Confirming this will require spatially resolved fermentation models followed by well-designed human studies.

This framework could inform functional-food development by placing greater emphasis on fibre structure and accessibility, rather than quantity alone.

## 9. Future Perspectives

The relationship between barley architecture and colonic fermentation discussed in this review raises three questions that existing methods can test:

Does structural preservation slow substrate utilisation? Intact plant cell walls restrict enzyme access to starch inside the cells; for example, purified cellulose can directly inhibit α-amylase activity, and the cell wall’s integrity decreases starch breakdown in preserved plant tissues compared to disrupted ones in kinetic assays [91]. However, barley endosperm cell walls are mainly composed of β-glucan and arabinoxylan, which remain underexplored. This is more significant in barley than in cereals with higher native resistant starch, since barley starch itself is only mildly resistant (Section 3.1). If most of the resistant fraction results from encapsulation rather than granule crystallinity, then processing that breaks the wall may substantially reduce this fraction. Consequently, the difference between coarse and fine formats should be correspondingly large. Grain structural properties may affect both small-intestinal digestion and the timing and location of fermentation [92]. Building on these observations, we hypothesise that encapsulated resistant starch and β-glucan in structurally preserved barley matrices may delay access by Tier 1 organisms and thereby postpone the downstream cross-feeding cascade involving Tier 3 butyrate producers. In contrast, in disrupted matrices like barley flour, degraders may allow earlier microbial access to substrates. Two studies support this idea: Lu et al. (2023) reported delayed starch utilisation in intact highland barley matrices, with significant butyrate production after 24–48 h in batch in vitro fermentations [10]. Similarly, Ntouranidi (2025) found that coarser barley husk slowed utilisation by human faecal microbiota compared to finer husk, with both particle size and composition playing roles [32]. Resolving this requires comparison of coarse and fine formats from the same batch under identical digestion and fermentation conditions and separating two mechanisms that both slow digestion but respond in opposite ways to processing: physical encapsulation, which may be disrupted by milling and other forms of mechanical breakdown, and molecular interaction between solubilised β-glucan and starch, which cooking may modulate. Measuring β-glucan solubility alongside starch hydrolysis kinetics would separate them.

Does structural preservation shift fermentation distally? A structurally preserved matrix could sustain substrate delivery further along the colon, extending the cross-feeding cascade into the distal region [86]. Human studies show that both the site and amount of SCFA delivery may matter in determining host hormonal signalling, as discussed in Section 6. Addressing this requires a spatially resolved model such as M-SHIME, with time-resolved SCFA measurement across the proximal, transverse and distal colon. Neither faecal endpoints nor single in vitro batch fermentations can resolve the site of fermentation (Section 5).

Do fermentation kinetics and transit time interact? A gap exists between studies that vary particle size while holding fermentation conditions constant and those that vary transit time while holding substrate constant. To our knowledge, no studies have examined the combined effects of fermentation kinetics and the distribution of butyrate, although Minnebo et al. (2023) [101] reported non-linearity. Linking the two would require fermentation of structurally defined barley formats under controlled retention times, followed by human studies that measure transit time in real time and treat it as a variable rather than a nuisance. Grain architecture could then potentially become a tractable variable in personalised nutrition.

Beyond these questions, the consequences of a modified fermentation site on host hormonal signalling remain open-ended. Regional differences in L-cell content and per-cell hormonal output (Section 6), together with human evidence that distal SCFA delivery alters fat oxidation and PYY [12], suggest that site of fermentation may have both endocrine and epithelial consequences. However, whether SCFA patterns created by structurally intact barley matrices are large enough to engage these pathways is largely unexplored. This will further require human studies that combine region-specific SCFA production with plasma hormone and appetite endpoints.

## Data Availability

No new data were created or analyzed in this study. Data sharing is not applicable to this article.

## Abbreviations

The following abbreviations are used in this manuscript:

CAZyme	Carbohydrate-active enzyme
DM	Dry mass
DP3	Degree of polymerisation 3 (cellotriose unit)
DP4	Degree of polymerisation 4 (cellotetraose unit)
FFAR2	Free fatty acid receptor 2
FFAR3	Free fatty acid receptor 3
GI	Glycaemic index
GLP-1	Glucagon-like peptide-1
INSL5	Insulin-like peptide 5
M-SHIME	Mucosal Simulator of the Human Intestinal Microbial Ecosystem
NSP	Non-starch polysaccharide
PUL	Polysaccharide utilisation locus
PYY	Peptide YY
RS	Resistant starch
RS1-RS5	Resistant starch types 1 to 5
SCFA	Short-chain fatty acid
TGR5	Takeda G protein-coupled receptor 5 (bile acid receptor GPBAR1)
